# Association between biological aging and diabetic retinopathy

**DOI:** 10.1038/s41598-024-60913-x

**Published:** 2024-05-02

**Authors:** Haoxian Tang, Nan Luo, Xuan Zhang, Jingtao Huang, Qinglong Yang, Hanyuan Lin, Xinyi Zhang

**Affiliations:** 1 Shantou University Medical College, Shantou, Guangdong China; 2 Department of Cardiology, The First Affiliated Hospital of Shantou University Medical College, Shantou, Guangdong China; 3 Department of Psychiatry, Shantou University Mental Health Center, Shantou, Guangdong China; 4 Department of Bone and Joint Surgery, Peking University Shenzhen Hospital, Shenzhen, Guangdong China; 5 Department of Sports Medicine and Rehabilitation, Peking University Shenzhen Hospital, Shenzhen, Guangdong China; 6 Department of Urology, The Second Affiliated Hospital of Shantou University Medical College, Shantou, Guangdong China; 7 Department of Ophthalmology, The First Affiliated Hospital of Shantou University Medical College, No. 57 Changping Road, Shantou, 515041 Guangdong China

**Keywords:** Biological aging, Chronological age, Diabetic retinopathy, NHANES, Diseases, Eye diseases, Retinal diseases, Pathogenesis

## Abstract

The impact of aging on diabetic retinopathy (DR) remains underestimated. The current study aimed to investigate the association between biological aging and DR, in contrast to chronological age (CA). Using the National Health and Nutrition Survey data from 2005 to 2008. Biological aging was evaluated through the biological age (BA) and phenotypic age (PA), which were calculated from clinical markers. DR was identified in participants with diabetes mellitus (DM) when they exhibited one or more retinal microaneurysms or retinal blot hemorrhages under retinal imaging, with or without the presence of more severe lesions. Survey-weighted multivariable logistic regression was performed, and the regression model was further fitted using restricted cubic splines. The discriminatory capability and clinical utility of the model were evaluated using receiver operating characteristic (ROC) curves and decision curve analysis (DCA). Based on weighted analyses, of the 3100 participants included in this study, of which 162 had DR. In the adjusted model, BA (odds ratio [OR] = 1.12, 95% CI, 1.06–1.18) and PA (OR = 1.11, 95% CI, 1.07–1.14) were associated with DR, while CA was not significantly (OR = 1.01, 95% CI, 0.99–1.03). Narrowing the analysis to DM participants and adjusting for factors like insulin showed similar results. ROC and DCA analyses indicate that BA/PA predicted DR better than CA and offer greater clinical utility. The positive association between BA/PA and DR was consistent across subgroups despite potential interactions. Biological aging heightens DR risk, with BA/PA showing a stronger association than CA. Our findings underscored the importance of timely anti-aging interventions for preventing DR.

## Introduction

Diabetic retinopathy (DR) is a prevalent microvascular complication of diabetes mellitus (DM) and remains the leading cause of avoidable blindness among individuals of working age^1^. The Global Burden of Disease study highlights the substantial prevalence and impact of DR^2^. While the age-standardized prevalence of blindness caused by factors like cataracts, glaucoma, refractive errors, and macular degeneration decreased between 1990 and 2020, DR stood out as an exception^2^. In 2020, DR resulted in over 2.9 million cases of moderate or worse vision impairment among adults aged 50 years and older^2^. The global population of individuals with DM is projected to exceed 780 million by 2045, while DR and related visual impairment are expected to affect approximately 160.5 million individuals^3,4^. Given that early detection and timely intervention can significantly prevent visual impairment and blindness associated with DR, it is crucial to prioritize the targeting of DR as a key focus for prevention and treatment efforts.

Aging entails gradual loss of physiological integrity, resulting in impaired function and heightened vulnerability to mortality^5^. The underestimated contribution of aging to DR development necessitates further investigation, despite shared risk factors identified in previous studies^6^. While chronological age (CA) is a powerful risk factor for aging-related diseases and mortality, individuals of the same age may experience different rates of biological aging and susceptibilities^7^. Considering the modifiability of biological aging, interventions to slow its progression, and the preventability of DR, distinguishing CA from physiological aging early in life is imperative for timely identification and intervention in at-risk individuals or groups^8^. Various methods, like DNA methylation age (DNAmA) and leukocyte telomere length, measure biological aging; however, they only capture a limited aspect of the comprehensive changes associated with the multifactorial process of aging^8^. In contrast, Klemera and Horvath et al.’s clinical biomarker-based measurements of biological aging (biological age [BA] and phenotypic age [PA]) capture multiple indicators of aging at the cellular and intracellular levels^9,10^, closely align with disease progression and individualized biological aging levels^7,11^, and serve as practical and reliable predictors of aging outcomes for large-scale implementation in public health surveillance settings^12^.

The current study aimed to investigate the association of biological aging (measured by BA and PA) with DR in a nationally representative sample and compare it with CA.

## Methods

### Data sources

The National Health and Nutrition Survey (NHANES) was a continuous cross-sectional survey conducted by the National Center for Health Statistics (NCHS), implementing a sophisticated stratified, multistage probability cluster sampling approach to holistically assess the health and behavioral patterns of the non-institutionalized U.S. population. Given that retinal imaging was exclusively undertaken on participants aged 40 years and older during NHANES 2005–2006 and 2007–2008, this study incorporated solely non-identifiable data from this subset of participants. The NHANES protocol obtained approval from the institutional review board at the NCHS, with all participants granting written informed consent upon enrollment. This study adhered to the revised 2013 Declaration of Helsinki. Utilizing publicly available de-identified data, the study qualifies for exemption from the requirement of informed consent in accordance with the applicable regulations of the Shantou University Medical College Institutional Review Board. The study followed the Strengthening the Reporting of Observational Studies in Epidemiology (STORBE) reporting guideline.

### Study design and population

**Figure 1:**
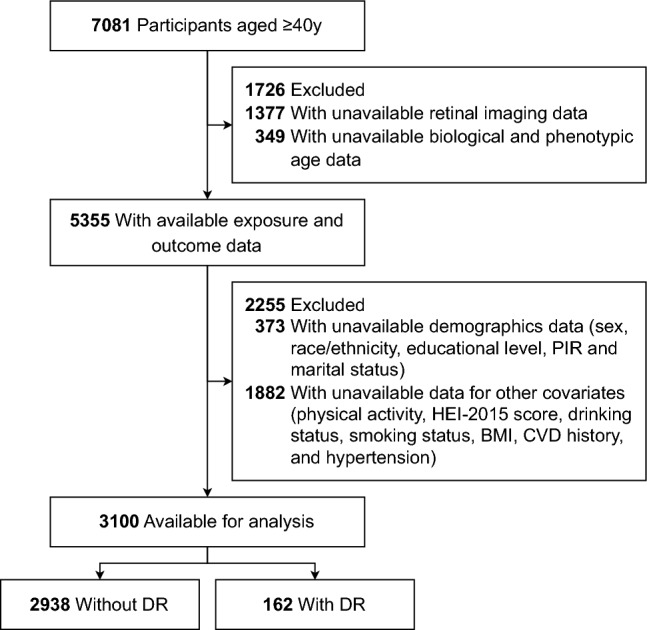
Flow diagram of the screening and enrollment of study participants. *BMI* body mass index, *CVD* cardiovascular disease, *DR* diabetic retinopathy, *HEI-2015* Healthy Eating Index-2015, *PIR* poverty income ratio.

As shown in Fig. 1, from an initial group of 7081 participants aged ≥ 40 years, we excluded 1726 due to unavailable retinal imaging and BA/PA data, and removed 373 with missing demographic information (including sex, race/ethnicity, educational level, poverty income ratio [PIR], and marital status). An additional 1882 were excluded due to missing data on other covariates (including physical activity, Healthy Eating Index-2015 [HEI-2015] score, drinking status, smoking status, body mass index [BMI], cardiovascular disease [CVD] history, and hypertension). The final analysis included 2938 participants without DR and 162 with DR.

### Assessment of biological aging

Biological Aging was evaluated through the assessment of BA and PA. BA and PA employ distinct computational algorithms and integrate diverse biomarkers to quantify the process of biological aging. BA and PA acceleration are defined as whether BA/PA is greater than CA.

The Klemera–Doubal method was employed to determine BA, relying on the assessment of 8 biomarkers: Ln-C-reactive protein (Ln-CRP), serum creatinine, glycosylated hemoglobin, serum albumin, serum total cholesterol, serum urea nitrogen, serum alkaline phosphatase, and systolic blood pressure^9,13,14^. Its calculation process mainly includes the following steps^13^.

Step 1: Construct a series of regressions of individual biomarkers on CA, and obtain the regression slope (*k*), intercept (*q*), root mean squared error (*s*), and variance explained (*r*^2^).

Step 2: Preliminary calculation of biological age based on the parameters from Step 1, where *j* denotes the number of biomarkers and *BA*_*E*_ represents the optimum estimate of BA in linear case.

1 $$B{A}_{E}=\frac{{\Sigma }_{\dot{J}=1}^{m}\left({x}_{\dot{J}}-{q}_{j}\right)\left(\frac{{k}_{j}}{{s}_{j}^{2}}\right)}{{\Sigma }_{j=1}^{m}{\left(\frac{{k}_{j}}{{s}_{j}}\right)}^{2}}$$

Step 3: Subsequent calculation of the characteristic correlation coefficient (*r*_*char*_) and scaling factor (*S*_*BA*_^2^) to consider the influence of CA in Eq. (1), where *i* denotes the number of samples.

2 $${r}_{char}=\frac{{\Sigma }_{j=1}^{m}\frac{{r}_{j}^{2}}{\sqrt{1-{r}_{j}^{2}}}}{{\Sigma }_{j=1}^{m}\frac{{r}_{j}}{\sqrt{1-{{r}_{j}}^{2}}}}$$

3 $${S}_{BA}^{2}=\frac{{\Sigma }_{j=1}^{n}{\left(\left(B{A}_{{E}_{j}}-C{A}_{j}\right)-\frac{{\Sigma }_{i=1}^{n}(B{A}_{{E}_{j}}-C{A}_{i})}{n}\right)}^{2}}{n}-\left(\frac{1-{r}_{char}^{2}}{{r}_{char}^{2}}\right)\times (\frac{{({CA}_{max}-{CA}_{min})}^{2}}{12m})$$

Step 4: Incorporate CA into Eq. (1) and calculate the final BA.

4 $$BA=\frac{{\Sigma }_{j=1}^{m}\left({x}_{j}-{q}_{j}\right)\left(\frac{{k}_{j}}{{s}_{j}^{2}}\right)+\frac{CA}{{S}_{BA}^{2}}}{{\sum }_{j=1}^{m}{\left(\frac{{k}_{j}}{{s}_{j}}\right)}^{2}+\frac{1}{{S}_{BA}^{2}}}$$

PA was determined through algorithms derived from multivariate analysis of mortality hazard^15^. These algorithms incorporated 10 aging-related variables, namely CA, albumin, creatinine, glucose, CRP, lymphocyte percent, mean cell volume, red blood cell distribution width, alkaline phosphatase, and white blood cell count^7^.

5 $$PA=141.50+\frac{Ln\left[-0.00553\times Ln({\text{exp}}(\frac{-1.51714\times {\text{exp}}(xb)}{0.0076927}))\right]}{0.09165}$$

xb = − 19.907 − 0.0336 × Albumin (g/L) + 0.0095 × Creatinine (μmol/L) + 0.1953 × Glucose (mmol/L) + 0.0954 × Ln-CRP (mg/dL) − 0.0120 × Lymphocyte Percent (%) + 0.0268 × Mean Cell Volume (fL) + 0.3306 × Red Cell Distribution Width (%) + 0.00188 × Alkaline Phosphatase (U/L) + 0.0554 × White Blood Cell Count (1000 cells/μL) + 0.0804 × CA (years)^13^.

### Diagnosis of DR

The diagnostic criteria for DM encompass a doctor’s diagnosis, glycohemoglobin (HbA1c) levels exceeding 6.5%, fasting glucose levels of 7.0 mmol/L or higher, random/2-h oral glucose tolerance test (OGTT) blood glucose levels reaching 11.1 mmol/L or above, or the utilization of DM medication/insulin^16^.

NHANES 2005–2008 utilized the Canon CR6-45NM ophthalmic digital imaging system and Canon EOS 10D digital camera (Canon, Tokyo, Japan) to capture two digital images per eye without pharmacological dilation of the pupils^17^. The digital images underwent grading using a modified version of the Airlie House classification scheme for retinopathy at the University of Wisconsin Ocular Epidemiologic Reading Center (Madison, WI)^18^. In cases where retinopathy severity could not be graded in one eye, an analogous grade was assigned based on the other eye. DR was identified in participants with DM when they exhibited one or more retinal microaneurysms or retinal blot hemorrhages, with or without the presence of more severe lesions, adhering to the grading standards set by the Early Treatment Diabetic Retinopathy Study (ETDRS)^19^.

### Covariates

Age, sex, race/ethnicity, PIR, marital status, education level, physical activity, HEI-2015 score, drinking status, smoking status, BMI, CVD history, and hypertension were deemed potential confounding variables. Self-reported race/ethnicity data was categorized into five distinct groups: Mexican American, non-Hispanic White, non-Hispanic Black, other Hispanic, and others, which encompassed individuals with multiracial backgrounds^16^. Marital status was classified into four categories: married, never married, living with a partner, and others, which included individuals who were widowed, divorced, or separated^16^. Education level was segmented into three tiers: less than high school, high school or equivalent, and above high school^16^. physical activity encompasses self-reported time spent on activities like walking, biking, household chores, work-related tasks, and recreation during the week. HEI-2015 score evaluated diet quality based on 13 components, summing to a 100-point score indicating adherence to 2015–2020 Dietary Guidelines^20^, with details in Table S1. Drinking status was categorized into five groups: never (consumed < 12 drinks in lifetime), former (< 12 drinks in lifetime, none in the past year), mild (≤ 1 drink per day for females, ≤ 2 drinks per day for males in the last 12 months), moderate (≤ 2 drinks per day for females, ≤ 3 drinks per day for males in the last 12 months), and heavy (≥ 3 drinks per day for females, ≥ 4 drinks per day for males in the last 12 months)^21^. Smoking status can be categorized into three groups: never (less than 100 cigarettes smoked in life), former (more than 100 cigarettes smoked but currently quit), and now (more than 100 cigarettes smoked and currently smoking^16^. BMI was calculated as the weight (in kilograms) divided by the square of the height (in meters)^22^. CVD history was documented as a history of being diagnosed with heart failure, coronary heart disease, angina, heart attack, or stroke. The average blood pressure is calculated, excluding zero diastolic readings unless all are zero; for a single reading, it serves as the average, and for multiple readings, the first is excluded. Hypertension is diagnosed when systolic is ≥ 140 mmHg or diastolic is ≥ 90 mmHg. Age, PIR, physical activity, HEI-2015 score, and BMI were treated as continuous variables in the model. Additionally, age was categorized as < 60, 60–69, and ≥ 70 when utilized as an exposure variable or considered in subgroup analyses.

### Statistical analysis

The NHANES employed a complex multi-stage probability sampling method. Each sample person was assigned a sample weight, which could be considered a measure of the number of individuals represented by that particular sample person. When data from NHANES were weighted, the sample was deemed representative of the U.S. civilian noninstitutionalized population. In our analyses, we incorporated the weights, clustering, and stratification information of the samples^23^.

Participant characteristics were calculated based on the presence or absence of DR. Categorical variables were presented as numbers (percentages, %), while continuous variables with a normal distribution were reported as means (Standard Error, SE). To analyze differences in characteristics across different patterns, Chi-squared test with Rao and Scott’s second-order correction (for categorical variables), Wilcoxon rank-sum test (for non-normally distributed continuous variables), and *t*-tests (for normally distributed continuous variables) were employed.

Traditional regression approaches may produce biased results, potentially leading to underestimated standard errors and confidence intervals, as well as an elevated risk of class I errors in hypothesis testing^24^. Aligning with recommendations from the existing literature, we adopted survey-weighted multivariable logistic regression, which fits a model to complex survey data, with inverse-probability weighting and design-based standard errors^24,25^.

Survey-weighted multivariable logistic regression was performed to assess associations of BA/PA/CA and BA/PA acceleration with DR. Model 1 was the crude model without adjustment for covariates. Model 2 was adjusted for age, sex, race/ethnicity, PIR, marital status, and education level. Model 3 was adjusted as for model 2, additionally adjusted for physical activity, HEI-2015 score, drinking status, smoking status, BMI, CVD history, and hypertension. Linear trend tests were conducted by treating categorical variables as continuous parameters. Splines were fit by a logistic regression model based on restricted cubic splines (3 knots at 10%, 50%, and 90%) and adjustments as used in Model 3.

Receiver operating characteristic (ROC) curves were used to assess the diagnostic value of BA/PA/CA for DR, with the area under the curve (AUC) measured by the C-statistic used to quantify predictive power. Decision curve analysis (DCA) was employed to evaluate the clinical utility of these models by estimating net benefits at various threshold probabilities.

Stratified analyses were performed based on age (< 60, 60–69, or ≥ 70 years), gender (female or male), race/ethnicity (Mexican American, non-Hispanic Black, non-Hispanic White, other Hispanic, or other), CVD history (yes or no), hypertension (yes or no), smoking status (never, former, or now), and drinking status (never, former, mild, moderate, or heavy). To test for interaction, a cross-product term was added to the regression model to examine the effect of one variable on the outcome based on the level of another variable^16^.

For sensitivity analyses, we restricted the study population to DM and repeated the regression analysis. Furthermore, considering potential confounding from different types of DM, we additionally adjusted for fasting insulin and the homeostasis model assessment of insulin resistance (HOMA-IR) to assess the robustness of the results. The calculation formula for HOMA-IR is as follows: fasting plasma glucose (mmol/L) × fasting insulin (μU/mL)/22.5^26^.

All analyses were conducted using R, version 4.2.2 (R Project for Statistical Computing), along with the survey package (version 4.2-1) and Free Software Foundation statistics software, version 1.9.2. Statistical significance was determined by two-sided *P* values below 0.05.

### Ethics approval and consent to participate

The National Health and Nutrition Examination Survey (NHANES) is conducted by the Centers for Disease Control and Prevention (CDC) and the National Center for Health Statistics (NCHS). The NCHS Research Ethics Review Committee reviewed and approved the NHANES study protocol. All participants signed written informed consent.

## Results

### Characteristics of the participants

Utilizing weighted analyses, the study encompassed 3100 participants, representing 70,772,414 individuals nationwide, with a weighted mean age of 55.53 years (SE, 0.45), with 1464 females (weighted percentage, 50.40%). 162 participants were diagnosed with DR. Participants in the DR group display higher CA, BA, and PA, with a higher proportion of males and non-Hispanic black, lower educational level, lower PIR, higher BMI, and former smoking or alcohol use. A notable proportion of participants within the DR group present a history of CVD or hypertension, as detailed in Table 1.

**Table 1:** Characteristics of participants in the NHANES 2005–2008 cycles.

Characteristics	Total (n = 3100)	Without DR (n = 2938)	With DR (n = 162)	*P* value
Weighted population	70,772,414	68,362,538	2,409,875	
Chronological age, mean (SE), years	55.53 (0.45)	55.38 (0.46)	59.97 (0.74)	< 0.001
Chronological age, no. (%)				0.01
< 60	1651 (67.07)	1587 (67.63)	64 (51.01)	
60–69	788 (19.30)	731 (18.98)	57 (28.52)	
≥ 70	661 (13.63)	620 (13.39)	41 (20.47)	
Sex, no. (%)				< 0.001
Female	1464 (50.40)	1405 (50.98)	59 (33.87)	
Male	1636 (49.60)	1533 (49.02)	103 (66.13)	
Race/ethnicity, no. (%)				< 0.001
Mexican American	431 (4.36)	405 (4.31)	26 (5.80)	
Non-Hispanic Black	525 (7.12)	470 (6.70)	55 (19.01)	
Non-Hispanic White	1875 (82.24)	1810 (82.72)	65 (68.61)	
Other Hispanic	177 (2.46)	164 (2.34)	13 (5.77)	
Other Race	92 (3.83)	89 (3.94)	3 (0.81)	
Marital status, no. (%)				0.66
Married	1976 (68.29)	1877 (68.39)	99 (65.50)	
Never married	202 (5.80)	194 (5.84)	8 (4.69)	
Living with partner	135 (4.44)	129 (4.47)	6 (3.75)	
Other	787 (21.46)	738 (21.30)	49 (26.06)	
Educational level, no. (%)				0.004
Less than high school	712 (22.97)	655 (13.83)	57 (25.94)	
High school or equivalent	759 (24.48)	716 (24.42)	43 (30.69)	
Above high school	1629 (52.55)	1567 (61.75)	62 (43.37)	
PIR, mean (SE)	3.51 (0.07)	3.52 (0.07)	3.11 (0.17)	0.01
Physical activity, mean (SE), min/week	664.16 (34.21)	663.70 (35.90)	677.25 (144.75)	0.34
HEI-2015 score, mean (SE)	55.30 (0.53)	55.23 (0.56)	57.20 (0.95)	0.10
BMI, mean (SE), kg/m^2^	28.75 (0.19)	28.64 (0.19)	31.90 (0.62)	< 0.001
Smoking status, no. (%)				0.02
Never	1458 (48.19)	1367 (47.73)	91 (61.21)	
Former	1048 (32.62)	999 (32.81)	49 (27.40)	
Now	594 (19.19)	572 (19.47)	22 (11.39)	
Drinking status, no. (%)				< 0.001
Never	364 (9.23)	352 (12.33)	10 (4.37)	
Former	732 (19.46)	341 (9.01)	23 (15.50)	
Mild	1198 (43.40)	1150 (43.67)	48 (35.86)	
Heavy	362 (12.05)	660 (18.72)	72 (40.36)	
Moderate	444 (15.85)	435 (16.27)	9 (3.90)	
CVD history, no. (%)				< 0.001
No	2704 (90.50)	2590 (91.15)	114 (71.96)	
Yes	396 (9.50)	348 (8.85)	48 (28.04)	
Hypertension				< 0.001
No	1503 (48.48)	1463 (54.26)	40 (31.49)	
Yes	1597 (51.52)	1475 (45.74)	122 (68.51)	
Biological age, mean (SE), years	54.85 (0.48)	54.46 (0.49)	65.74 (1.05)	< 0.001
Biological age acceleration, no. (%)				< 0.001
No	1822 (60.16)	1781 (61.34)	41 (26.88)	
Yes	1278 (39.84)	1157 (38.66)	121 (73.12)	
Phenotypic age, mean (SE), years	51.29 (0.50)	50.74 (0.51)	67.00 (1.31)	< 0.001
Phenotypic age acceleration, no. (%)				< 0.001
No	2396 (81.02)	2336 (82.58)	60 (36.86)	
Yes	704 (18.98)	602 (17.42)	102 (63.14)	

*BMI* body mass index, *CVD* cardiovascular disease, *DR* diabetic retinopathy, *HEI-2015* Healthy Eating Index-2015, *PIR* poverty income ratio, *SE* standard error. All means and SEs for continuous variables and percentages for categorical variables were weighted.

A total of 598 participants were diagnosed with DM, of which the prevalence of DR was 27.1%. Additionally, among DM participants, CA showed no significant difference between DR and non-DR individuals, while BA and PA were significantly higher in the DR group (Supplementary Table S2).

### Association of BA/PA/CA and BA/PA acceleration with DR

**Figure 2:**
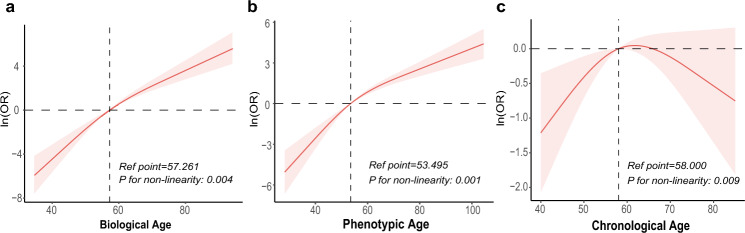
Association of biological, phenotypic, and chronological age with diabetic retinopathy. Data were fit by a survey-weighted multivariable logistic regression model based on restricted cubic splines. Data were adjusted for age, sex, race/ethnicity, PIR, marital status, education level, physical activity, HEI-2015 score, drinking status, smoking status, BMI, CVD history, and hypertension (Model 3). Age was not adjusted for in the regression model for chronological age.

Table 2 presents the results of the sample-weighted multivariable logistic regression analysis examining the association between BA/PA/CA and DR. In the crude model, each one-year increase in PA was associated with a 7% higher risk of DR, while a one-year increase in BA/CA was associated with a 4% higher DR risk. After adjusting for confounding factors (Model 3), the association between BA/PA and DR persisted, revealing a non-linear trend (Fig. 2). Participants who experienced aging acceleration had a significantly higher risk of developing DR compared with those who did not (BA acceleration: OR = 3.80, 95% CI, 2.01–7.18; PA acceleration: OR = 6.52, 95% CI, 3.45–12.32). Conversely, the association between CA and DR lost its statistical significance (OR = 1.01, 95% CI, 0.99–1.03). Upon further stratification based on CA (< 60, 60–69, and ≥ 70), no increased risk of DR was observed in older age brackets compared to younger ones.

**Table 2:** Association of biological, phenotypic, and chronological age with diabetic retinopathy.

	Model 1		Model 2		Model 3	
	OR (95% CI)	*P* value	OR (95% CI)	*P* value	OR (95% CI)	*P* value
Biological age	1.04 (1.02–1.06)	< 0.001	1.10 (1.06–1.14)	< 0.001	1.12 (1.06–1.18)	0.001
Biological age acceleration						
No	1 [Reference]		1 [Reference]		1 [Reference]	
Yes	4.32 (2.76–6.76)	< 0.001	4.35 (2.59–7.31)	< 0.001	3.80 (2.01–7.18)	0.002
Phenotypic age	1.07 (1.06–1.09)	< 0.001	1.12 (1.09–1.14)	< 0.001	1.11 (1.07–1.14)	< 0.001
Phenotypic age acceleration						
No	1 [Reference]		1 [Reference]		1 [Reference]	
Yes	8.12 (5.36–12.30)	< 0.001	7.00 (4.36–11.25)	< 0.001	6.52 (3.45–12.32)	< 0.001
Chronological age	1.04 (1.02–1.05)	< 0.001	1.03 (1.02–1.05)	< 0.001	1.01 (0.99–1.03)	0.19
Subgroups						
< 60	1 [Reference]		1 [Reference]		1 [Reference]	
60–69	1.99 (1.09–3.66)	0.03	1.94 (1.02–3.69)	0.04	1.34 (0.66–2.72)	0.36
≥ 70	2.03 (1.38–2.97)	< 0.001	1.85 (1.23–2.78)	0.01	1.06 (0.60–1.89)	0.81
Trend test		< 0.001		0.003		0.64

*CI* confidence interval, *OR* odd ratio. Model 1 was the crude model without adjustment for covariates. Model 2 was adjusted for age, sex, race/ethnicity, PIR, marital status, education level. Model 3 was adjusted as for model 2, additionally adjusted for physical activity, HEI-2015 score, drinking status, smoking status, BMI, CVD history, and hypertension. Age was not adjusted for in the regression model for chronological age.

**Figure 3:**
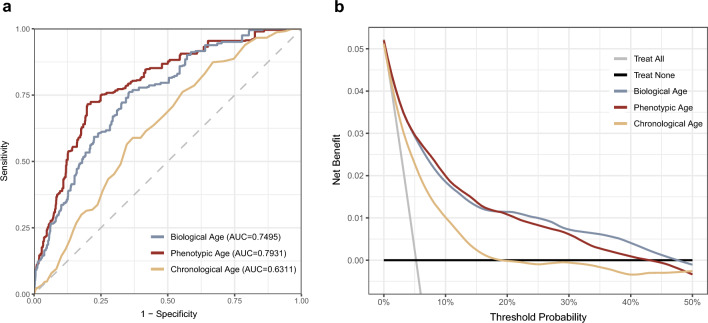
Receiver operating characteristic (ROC) curves, the area under curve (AUC), and decision curve analysis (DCA) of biological, phenotypic, and chronological age.

The ROC curve shows that BA (AUC = 0.7495) and PA (AUC = 0.7931) outperform CA (AUC = 0.6311) in predicting DR (Fig. 3a). DCA indicates that when the threshold is below 50%, BA/PA has a higher net benefit than CA (Fig. 3b).

### Subgroup and sensitivity analyses

Interactions of age with BA, and sex with PA were observed (*P* for interaction < 0.05). However, a consistent positive association of BA/PA with DR was observed in all subgroups (Supplementary Table S3). It is noteworthy that a significant positive correlation between CA and DR was observed among participants aged < 60 (OR = 1.08, 95% CI, 1.01–1.15), as compared to the age groups of 60–69 and ≥ 70 years, despite the absence of significant interaction effects.

Restricting the analysis to DM patients produced results consistent with previous findings (Supplementary Table S4). Upon accounting for insulin secretion and resistance, the associations between BA/PA/CA and DR remained consistent with our conclusions (Supplementary Table S5).

## Discussion

In this nationally representative cross-sectional study, after adjusting for confounding factors such as demographics, lifestyle, and medical history, participants with higher BA/PA were found to have a higher risk of DR, while the association between CA and DR was not significant. ROC curves and DCA suggest BA/PA outperforms CA in predicting DR, with higher clinical utility. Despite potential interactions, the positive association between BA/PA and DR remained consistent across subgroups. When narrowing the analysis to DM participants and accounting for confounding factors like insulin secretion and resistance, our conclusion remained unchanged.

The aging process impacts the retina in various ways. Mohamed et al. discovered that the overall retinal thickness decreased in male albino rats as they aged, particularly in the inner nuclear layer (INL)^27^. A similar trend was observed by Barboni et al. using optical coherence tomography (OCT) imaging in both dominant optic atrophy (DOA) patients and healthy individuals, with a decline in the retinal nerve fiber layer (RNFL) thickness as they aged^28^. Furthermore, aging is linked to reduced retinal macular blood flow^29^, slower motility and injury response of retinal microglia, increased cell density, and smaller dendritic spindles^30^, as well as heightened susceptibility of Müller cells to oxidative stress^31^.

Aging, characterized by hallmarks such as genomic instability, epigenetic alterations, telomere attrition, mitochondrial dysfunction, and cellular senescence^5^, impacts cellular function. The interplay between aging and the progression of DR encompasses intricate mechanisms. In DR, senescent cells accumulate in the retina, exacerbated by DM-induced acceleration of aging and inflammatory pathways^32,33^. Retinal pigment epithelial cell damage and impaired immune responses further contribute^34^, potentially explaining the increased risk of DR with age. Oxidative stress and aging-related changes in autophagy also play roles in retinopathy’s progression in older individuals^6,35^.

While CA is considered an important risk factor for aging-related diseases, including DR, it may not accurately predict the occurrence of DR. In an Iranian study, the prevalence of DR increased with age from 1.0% in the 55–59 years group, peaking at 8.2% in the 70–74 years group^36^. However, for participants aged ≥ 75 years, the prevalence of DR was 2.4% and did not show further increases. Similarly, a study by Zhang et al. based on NHANES data found no significant difference in the prevalence of DR between individuals aged 40–64 years and those aged ≥ 65 years^37^. The multivariable logistic regression analysis also showed a non-significant association between CA and DR (OR = 0.99, 95% CI, 0.95–1.02), which is consistent with our findings. Furthermore, subgroup analysis in the current study revealed a significant positive association between CA and DR in participants aged < 60 years, but not in those aged 60–69 and ≥ 70 years. These results may seem contradictory to the concept of DR as an age-related disease, but they can be explained by the variability in biological aging among individuals of the same age. BA reflects an individual’s physiological condition, and can differ from their CA due to factors like genetics, lifestyle, and overall health. This variability in biological aging can lead to differences in susceptibility to aging-related diseases, including DR. Thus, while CA is an important risk factor for aging-related diseases, its impact on disease occurrence and progression may be influenced by individual differences in biological aging, highlighting the need for personalized approaches to disease management.

Zhu et al. utilized retinal fundus and OCT imaging from 11,052 disease-free participants in the UK Biobank to develop a deep learning model for retinal age estimation, defining the difference between retinal age and CA as the retinal age gap (RAG). Their research suggested a J-shaped relationship between RAG and all-cause mortality, indicating a significant threshold effect; when RAG exceeded 0, the risk of death began to slightly increase (HR = 1.01). However, the association between RAG and CVD or cancer mortality was not significant. Furthermore, subgroup analyses revealed that the association between RAG and all-cause mortality was not significant among individuals with DM^38^. Building on the conceptual framework established by Zhu et al., Gonzalez et al. employed a similar approach to model and analyze 13,544 fundus images, including 7694 images from DM patients without DR and 5850 images from DM patients with DR. They observed that the RAG in DM patients with DR was consistently higher than in those without DR, with RAG increasing with the severity of DR. However, this study was limited to a descriptive analysis of RAG, as the authors did not further investigate potential confounding factors^39^. Building upon Zhu et al.’s model, Chen et al. further analyzed 2311 DM patients and found that for each additional year of the RAG, the risk of DR increased by 7%. They grouped RAG into quartiles based on percentage and found a significantly higher prevalence of DR only in the highest quartile compared to the lowest quartile, suggesting a possible non-linear relationship. However, Chen et al. did not further fit the model using RCS^40^.

The RAG concept proposed by Zhu et al. and the BA/PA concept in the current study shared similarities, providing different perspectives on aging with their strengths and limitations. For example, RAG evaluated aging from retinal imaging, while BA/PA was based on biochemical markers in the blood. Additionally, RAG was computed using a deep learning model, which achieved higher precision but sacrificed some interpretability. On the other hand, BA/PA in this study was based on regression equations from different dimensions, offering higher interpretability but potentially lower precision compared to the deep learning model. Furthermore, as previously mentioned, aging is a complex process, and the RAG concept did not take into account the roles of various bodily systems in aging. Also, as demonstrated in the series of studies by Zhu et al., there was a significant association between RAG and CVD, stroke, kidney failure, obesity, metabolic syndrome, and inflammation^41–44^, and it was also found that cardiovascular health and blood glucose status had an impact on RAG^45,46^, all of which were potential or important pathways for the progression of biological aging. In contrast, BA/PA considered the distinct rates of aging across various bodily systems (such as metabolism, the immune system, liver, kidneys, etc.) and their respective contributions to the overall aging process at its conceptual stage. For instance, it took into account factors like albumin and glucose, which Putin and Mamoshina had highlighted as the most important blood biochemical predictors of biological aging through different methods and sample populations^47,48^. However, the algorithm of BA/PA did not consider biomarkers specific to the eyes.

The conceptualization and quantification of the RAG were groundbreaking but warranted further consideration. For instance, Zhu et al.’s series of studies indicated that the association patterns between RAG and health outcomes were mostly significant only in the high quartile, often suggesting non-linear relationships and threshold effects. However, Zhu et al. did not utilize RCS to visualize non-linear relationships in all studies. While some of Zhu et al.’s studies compared the predictive performance of RAG and traditional risk factors using ROC analysis, none found a significant difference in the AUC between RAG and traditional risk factors^41,49^. Additionally, Chen et al.’s study did not further compare the predictive performance of RAG and CA for DR. However, our study, as indicated by ROC curves and DCA, suggested that BA and PA could better predict DR compared to CA, with higher clinical utility. Since the inception of BA/PA, their ability to identify aging and predict diseases has been extensively validated. In contrast, the concept of RAG was still in its nascent stage, requiring further validation and optimization to enhance model interpretability and representativeness. Additionally, efforts should be made to increase RAG’s sensitivity to individualized biological aging.

The findings of the current study were not in conflict with those of Zhu, Gonzalez, and Chen but rather complemented each other. In the future, further discussion is needed to explore the differences between RAG and BA/PA from various perspectives, including their ability to identify aging, clinical utility, and public health implications. Additionally, further exploration of biomarkers closely associated with DR is warranted, examining the relationship between biological aging and DR from a multi-system, multi-omics perspective.

BA and PA assessment offers several key advantages. It is easily understood by the general population, unlike complex medical tests, making it a valuable tool for health education. Knowing their BA/PA can provide individuals with insights into their overall health and potential risks, empowering them to make informed decisions about their health and adopt healthier lifestyles. In clinical practice, BA/PA assessment using blood-derived biomarkers aligns with standard procedures, making it easy to integrate into routine healthcare. This assessment allows healthcare professionals to personalize interventions and treatments, improving the precision and effectiveness of care. Additionally, monitoring changes in BA/PA over time can help identify trends indicating increased disease risk, enabling early intervention and potentially preventing disease onset or progression. By prioritizing resources based on biological aging rather than CA, BA/PA assessment can change public health, providing a more accurate and personalized approach to health management.

Through meticulous quality control procedures and sophisticated sampling techniques employed in the NHANES dataset, the collected data provides a representative insight into the connection between biological aging and DR among U.S. adults. However, it is essential to acknowledge certain limitations. First, due to the cross-sectional study, we could not establish a definitive temporal relationship between biological aging and DR. Second, the possibility of measurement errors and recall bias cannot be completely avoided. Third, despite considering demographic characteristics, lifestyle factors, and specific medical conditions, the presence of potential confounding variables cannot be entirely excluded. Finally, although a large portion of the study sample consists of non-Hispanic white individuals, subgroup analyses showed consistent associations between BA/PA/CA and DR, regardless of ethnicity, with no significant interactions. This suggests the results may be generalizable beyond non-Hispanic whites. However, since this study only used U.S. samples, further research in diverse populations worldwide is warranted.

## Conclusion

BA and PA are more accurate in identifying DR Risk than CA. Our results confirm the significance of aging in DR development and underscore the preventive potential of early detection and timely anti-aging interventions.

### Supplementary Information

Supplementary Tables.

## Data Availability

The National Health and Nutrition Examination Survey data are publicly available at https://wwwn.cdc.gov/nchs/nhanes which is publicly available.
