# Supplementary material for: Association between biological aging and diabetic retinopathy
Source: Sci Rep. 2024 May 2;14:10123. doi: 10.1038/s41598-024-60913-x (PMC11065862; doi:10.1038/s41598-024-60913-x)
Supplement: Supplementary file 1 — Supplementary Tables. [file 41598_2024_60913_MOESM1_ESM.docx]

**Supplementary Materials Files**

To: **Association between Biological Aging and Diabetic Retinopathy: Advancing Beyond Chronological Age**

by Haoxian Tang, Nan Luo, Xuan Zhang, Jingtao Huang, Qinglong Yang, Hanyuan Lin, Xinyi Zhang

Supplementary Table S1. Healthy Eating Index-2015 components, point values, and standards for scoring

Supplementary Table S2. Characteristics of Participants with Diabetes Mellitus in the NHANES 2005-2008 Cycles

Supplementary Table S3. Association of Biological, Phenotypic, and Chronological Age with Diabetic Retinopathy among Different Subgroups

Supplementary Table S4. Association of Biological, Phenotypic, and Chronological Age with Diabetic Retinopathy in Participants with Diabetes Mellitus

Supplementary Table S6. Association of Biological, Phenotypic, and Chronological Age with Diabetic Retinopathy, after further adjustment for fasting insulin and HOMA-IR

**Supplementary Table S1. Healthy Eating Index-2015 components, point values, and standards for scoring**

| **Component** | **Maximum points** | **Standard for maximum score** | \|  \| \| --- \|   **Standard for minimum score of zero** |
| --- | --- | --- | --- | --- |
| **Adequacy** |  |  |  |
| Total Fruits | 5 | ≥0.8 c equivalents/1,000 kcal | No fruit |
| Whole Fruits | 5 | ≥0.4 c equivalents/1,000 kcal | No whole fruit |
| Total Vegetables | 5 | ≥1.1 c equivalents/1,000 kcal | No vegetables |
| Greens and Beans | 5 | ≥0.2 c equivalents/1,000 kcal | No dark green vegetables or beans and peas |
| Whole Grains | 10 | ≥1.5 oz equivalents/1,000 kcal | No whole grains |
| Dairy | 10 | ≥1.3 c equivalents/1,000 kcal | No dairy |
| Total Protein Foods | 5 | ≥2.5 oz equivalents/1,000 kcal | No protein foods |
| Seafood and Plant Proteins | 5 | ≥0.8 c equivalents/1,000 kcal | No seafood or plant proteins |
| Fatty Acids | 10 | (PUFAs^a^+MUFAs^b^)/SFAs^c^ ≥2.5 | (PUFAs+MUFAs)/SFAs ≤1.2 |
| **Moderation** |  |  |  |
| Refined Grains | 10 | ≤1.8 oz equivalents/1,000 kcal | ≥4.3 oz equivalents/1,000 kcal |
| Sodium | 10 | ≤1.1 g/1,000 kcal | ≥2.0 g/1,000 kcal |
| Added Sugars | 10 | ≤6.5% of energy | ≥26% of energy |
| Saturated Fats | 10 | ≤8% of energy | ≥16% of energy |

^a^ PUFAs=polyunsaturated fatty acids.

^b^ MUFAs=monounsaturated fatty acids.

^c^ SFAs=saturated fatty acids.

**Reference:** Reedy, J. et al. Evaluation of the Healthy Eating Index-2015. J Acad Nutr Diet 118, 1622–1633 (2018).

**Supplementary Table S2. Characteristics of Participants with Diabetes Mellitus in the NHANES 2005-2008 Cycles**

| **Characteristics** | **Total (N=598)** | **Non-DR(N=436)** | **DR(N=162)** | ***P* value** |
| --- | --- | --- | --- | --- |
| Weighted population | 9,863,245 | 7,453,370 | 2,409,875 |  |
| Chronological age, mean (SE), years | 59.55(0.54) | 59.42(0.69) | 59.97(0.74) | 0.62 |
| Chronological age, No. (%) |  |  |  | 0.99 |
| <60 | 234(51.12) | 170(51.15) | 64(51.01) |  |
| 60-69 | 205(28.57) | 148(28.58) | 57(28.52) |  |
| ≥70 | 159(20.32) | 118(20.27) | 41(20.47) |  |
| Sex, No. (%) |  |  |  | <0.001 |
| Female | 268(44.82) | 209(49.14) | 59(33.87) |  |
| Male | 330(55.18) | 227(50.86) | 103(66.13) |  |
| Race/ethnicity, No. (%) |  |  |  | 0.01 |
| Mexican American | 109(6.57) | 83(6.81) | 26(5.80) |  |
| Non-Hispanic Black | 152(13.04) | 97(11.11) | 55(19.01) |  |
| Non-Hispanic White | 277(73.23) | 212(74.72) | 65(68.61) |  |
| Other Hispanic | 49(4.70) | 36(4.35) | 13(5.77) |  |
| Other Race | 11(2.47) | 8(3.01) | 3(0.81) |  |
| Marital status, No. (%) |  |  |  | 0.79 |
| Married | 377(69.29) | 278(70.51) | 99(65.50) |  |
| Never married | 29(4.47) | 21(4.39) | 8(4.69) |  |
| Living with partner | 20(2.95) | 14(2.69) | 6(3.75) |  |
| Other | 172(23.29) | 123(22.40) | 49(26.06) |  |
| Educational level, No. (%) |  |  |  | 0.22 |
| Less than high school | 190(19.13) | 133(16.92) | 57(25.94) |  |
| High school or equivalent | 169(30.67) | 126(30.67) | 43(30.69) |  |
| Above high school | 239(50.20) | 177(52.41) | 62(43.37) |  |
| PIR, mean (SE) | 3.27(0.09) | 3.33(0.08) | 3.11(0.17) | 0.17 |
| Physical activity, mean (SE), min/week | 592.32(73.41) | 564.86(70.95) | 677.25(144.75) | 0.62 |
| HEI-2015 score, mean (SE) | 55.37(0.60) | 54.78(0.79) | 57.20(0.95) | 0.08 |
| BMI, mean (SE), kg.m^2^ | 32.34(0.27) | 32.48(0.32) | 31.90(0.62) | 0.41 |
| Smoking status, No. (%) |  |  |  | 0.01 |
| Never | 281(48.20) | 190(44.00) | 91(61.21) |  |
| Former | 229(37.97) | 180(41.39) | 49(27.40) |  |
| Now | 88(13.82) | 66(14.61) | 22(11.39) |  |
| Drinking status, No. (%) |  |  |  | 0.01 |
| Never | 90(13.69) | 67(13.11) | 23(15.50) |  |
| Former | 201(29.87) | 129(26.47) | 72(40.36) |  |
| Mild | 187(35.26) | 139(35.06) | 48(35.86) |  |
| Moderate | 67(13.11) | 58(16.09) | 9(3.90) |  |
| Heavy | 53(8.07) | 43(9.26) | 10(4.37) |  |
| CVD history, No. (%) |  |  |  | 0.03 |
| No | 463(78.87) | 349(81.10) | 114(71.96) |  |
| Yes | 135(21.13) | 87(18.90) | 48(28.04) |  |
| Hypertension |  |  |  | 0.92 |
| No | 178(31.11) | 138(30.99) | 40(31.49) |  |
| Yes | 420(68.89) | 298(69.01) | 122(68.51) |  |
| Biological age, mean (SE), years | 62.19(0.63) | 61.04(0.77) | 65.74(1.08) | <0.001 |
| Biological age acceleration, No. (%) |  |  |  | 0.02 |
| No | 229(36.70) | 188(39.88) | 42(26.88) |  |
| Yes | 369(63.30) | 248(60.12) | 121(73.12) |  |
| Phenotypic age, mean (SE), years | 62.12(0.84) | 60.54(1.05) | 67.00(1.31) | <0.001 |
| Phenotypic age acceleration, No. (%) |  |  |  | 0.01 |
| No | 308(49.79) | 248(53.96) | 60(36.86) |  |
| Yes | 290(50.21) | 188(46.04) | 102(63.14) |  |

Abbreviations: BMI, body mass index; CVD, cardiovascular disease; DR, diabetic retinopathy; HEI-2015, Healthy Eating Index-2015; PIR, poverty income ratio; SE, standard error. All means and SEs for continuous variables and numbers and percentages for categorical variables were weighted.

**Supplementary Table S3. Association of Biological, Phenotypic, and Chronological Age with Diabetic Retinopathy among Different Subgroups**

|  | **Biological age** | | **Phenotypic age** | | **Chronological Age** | |
| --- | --- | --- | --- | --- | --- | --- |
|  | **OR(95%CI)** | ***P* value** | **OR(95%CI)** | ***P* value** | **OR(95%CI)** | ***P* value** |
| Age |  | **0.022^*^** |  | 0.295^*^ |  | 0.13^*^ |
| <60 | 1.28(1.19-1.37) | <0.001 | 1.12(1.07-1.18) | <0.001 | 1.08(1.02-1.15) | 0.01 |
| 60-69 | 1.21(1.10-1.34) | 0.001 | 1.13(1.08-1.19) | <0.001 | 1.00(0.83-1.19) | 0.98 |
| ≥70 | 1.11(1.03-1.19) | 0.01 | 1.11(1.06-1.16) | <0.001 | 0.95(0.85-1.07) | 0.37 |
| Sex |  | 0.285^*^ |  | **0.034^*^** |  | 0.846^*^ |
| Female | 1.26(1.14-1.39) | <0.001 | 1.17(1.09-1.25) | <0.001 | 0.99(0.88-1.10) | 0.81 |
| Male | 1.16(1.09-1.24) | <0.001 | 1.10(1.07-1.13) | <0.001 | 1.09(1.01-1.17) | 0.04 |
| Race/ethnicity |  | 0.67^*^ |  | 0.688^*^ |  | 0.705^*^ |
| Non-Hispanic White | 1.17(1.10-1.25) | <0.001 | 1.12(1.07-1.16) | <0.001 | 1.04(0.97-1.12) | 0.22 |
| Not non-Hispanic White | 1.23(1.17-1.29) | <0.001 | 1.11(1.07-1.15) | <0.001 | 1.04(0.97-1.12) | 0.23 |
| CVD history |  | 0.10^*^ |  | 0.091^*^ |  | 0.44^*^ |
| No | 1.22(1.15-1.30) | <0.001 | 1.12(1.08-1.17) | <0.001 | 1.05(0.99-1.12) | 0.1 |
| Yes | 1.15(1.07-1.24) | 0.002 | 1.09(1.04-1.14) | 0.003 | 1.00(0.89-1.13) | 0.95 |
| Hypertension |  | 0.748^*^ |  | 0.884^*^ |  | 0.206^*^ |
| No | 1.34(1.19-1.51) | <0.001 | 1.16(1.09-1.23) | <0.001 | 1.04(0.95-1.14) | 0.35 |
| Yes | 1.16(1.11-1.22) | <0.001 | 1.10(1.07-1.14) | <0.001 | 1.03(0.97-1.10) | 0.27 |
| Smoking status |  | 0.749^*^ |  | 0.370^*^ |  | 0.302^*^ |
| Never | 1.19(1.12-1.27) | <0.001 | 1.10(1.05-1.15) | 0.001 | 1.03(0.97-1.09) | 0.3 |
| Former | 1.16(1.03-1.31) | 0.02 | 1.15(1.08-1.22) | <0.001 | 1.04(0.92-1.17) | 0.49 |
| Now | 1.34(1.19-1.51) | <0.001 | 1.14(1.07-1.21) | 0.001 | 1.09(0.95-1.25) | 0.2 |
| Drinking status |  | 0.242^*^ |  | 0.307^*^ |  | 0.379^*^ |
| Never | 1.27(1.09-1.46) | 0.004 | 1.11(1.01-1.22) | 0.03 | 1.08(0.94-1.23) | 0.27 |
| Former | 1.13(1.07-1.18) | <0.001 | 1.08(1.04-1.13) | <0.001 | 0.97(0.92-1.02) | 0.24 |
| Mild | 1.25(1.10-1.42) | 0.002 | 1.15(1.08-1.22) | <0.001 | 1.09(0.98-1.21) | 0.11 |
| Moderate | -^#^ | -^#^ | -^#^ | -^#^ | 1.04(0.94-1.16) | 0.39 |
| Heavy | 1.32(0.97-1.78) | 0.07 | 1.38(1.02-1.85) | 0.04 | 1.24(1.05-1.45) | 0.01 |

^*^ *P* for interaction. ^#^ OR and its confidence interval could not be calculated due to small sample size, covariates, or number of people with outcome events. Abbreviations: CI, Confidence interval; CVD, cardiovascular disease; DR, diabetic retinopathy; HEI-2015, Healthy Eating Index-2015; OR, Odd Ratio; PIR, poverty income ratio. All models were adjusted for age, sex, race/ethnicity, PIR, marital status, education level, physical activity, HEI-2015 score, drinking status, smoking status, BMI, CVD history, and hypertension, except for covariates used for subgrouping. Age was not adjusted for in the regression model for chronological age.

**Supplementary Table S4. Association of Biological, Phenotypic, and Chronological Age with Diabetic Retinopathy in Participants with Diabetes Mellitus**

|  | **Model 1** | | **Model 2** | | **Model 3** | |
| --- | --- | --- | --- | --- | --- | --- |
|  | **OR(95%CI)** | ***P* value** | **OR(95%CI)** | ***P* value** | **OR(95%CI)** | ***P* value** |
| **Biological age** | 1.07(1.06-1.09) | <0.001 | 1.18(1.13-1.25) | <0.001 | 1.19(1.12-1.26) | <0.001 |
| **Biological age acceleration** |  |  |  |  |  |  |
| No | 1[Reference] |  | 1[Reference] |  | 1[Reference] |  |
| Yes | 1.80(1.08-3.01) | 0.02 | 1.79(0.99-3.23) | 0.054 | 2.07(0.999-4.28) | 0.050 |
| **Phenotypic age** | 1.03(1.02-1.05) | <0.001 | 1.05(1.02-1.08) | <0.001 | 1.06(1.03-1.09) | 0.002 |
| **Phenotypic age** **acceleration** |  |  |  |  |  |  |
| No | 1[Reference] |  | 1[Reference] |  | 1[Reference] |  |
| Yes | 2.01(1.18-3.41) | 0.01 | 1.90(0.99-3.63) | 0.053 | 2.57(1.26-5.25) | 0.02 |
| **Chronological age** | 1.00(0.99-1.02) | 0.6 | 1.00(0.98-1.02) | 0.94 | 0.99(0.96-1.02) | 0.37 |
| Subgroups |  |  |  |  |  |  |
| <60 | 1[Reference] |  | 1[Reference] |  | 1[Reference] |  |
| 60-69 | 1.00(0.55-1.84) | >0.99 | 1.00(0.53-1.88) | 0.99 | 0.82(0.39-1.74) | 0.56 |
| ≥70 | 1.01(0.68-1.51) | 0.95 | 0.88(0.58-1.33) | 0.53 | 0.62(0.33-1.18) | 0.12 |
| Trend test |  | 0.96 |  | 0.63 |  | 0.14 |

Abbreviations: CI, Confidence interval; OR, Odd Ratio. Model 1 was the crude model without adjustment for covariates. Model 2 was adjusted for age, sex, race/ethnicity, PIR, marital status, education level. Model 3 was adjusted as for model 2, additionally adjusted for physical activity, HEI-2015 score, drinking status, smoking status, BMI, CVD history, and hypertension. Age was not adjusted for in the regression model for chronological age.

**Supplementary Table S6. Association of Biological, Phenotypic, and Chronological Age with Diabetic Retinopathy, after further adjustment for fasting insulin and HOMA-IR**

|  | **Model 4** | | **Model 5** | | **Model 6** | |
| --- | --- | --- | --- | --- | --- | --- |
|  | **OR(95%CI)** | ***P* value** | **OR(95%CI)** | ***P* value** | **OR(95%CI)** | ***P* value** |
| **Total Population (n=1486)** |  |  |  |  |  |  |
| Biological age | 1.15(1.09-1.22) | 0.001 | 1.14(1.08-1.21) | 0.001 | 1.13(1.06-1.20) | 0.003 |
| Phenotypic age | 1.11(1.06-1.16) | 0.001 | 1.10(1.05-1.15) | 0.002 | 1.09(1.04-1.14) | 0.005 |
| Chronological age | 1.01(0.99-1.04) | 0.34 | 1.01(0.98-1.04) | 0.38 | 1.01(0.98-1.04) | 0.50 |
| **Participants with Diabetes Mellitus (n=338)** |  |  |  |  |  |  |
| Biological age | 1.10(1.04-1.16) | 0.01 | 1.09(1.03-1.16) | 0.01 | 1.08(1.01-1.16) | 0.03 |
| Phenotypic age | 1.06(1.02-1.10) | 0.01 | 1.06(1.02-1.10) | 0.02 | 1.05(1.00-1.10) | 0.054 |
| Chronological age | 0.98(0.95-1.02) | 0.27 | 0.98(0.94-1.02) | 0.27 | 0.98(0.94-1.02) | 0.30 |

Abbreviations: CI, Confidence interval; OR, Odd Ratio; homeostatic model assessment of insulin resistance, HOMA-IR. After further excluding participants with missing data on fasting insulin and HOMA-IR, a total of 1,486 individuals were included in the analysis, of whom 338 were diagnosed with diabetes mellitus and 90 with diabetic retinopathy. Model 4 was adjusted for age, sex, race/ethnicity, PIR, marital status, education level, physical activity, HEI-2015 score, drinking status, smoking status, BMI, CVD history, hypertension, and fasting insulin. Model 5 was adjusted for age, sex, race/ethnicity, PIR, marital status, education level, physical activity, HEI-2015 score, drinking status, smoking status, BMI, CVD history, hypertension, and HOMA-IR. Model 6 was adjusted as for model 5, additionally adjusted for fasting insulin. Age was not adjusted for in the regression model for chronological age.
